# Structure of Polaronic Centers in Proton-Intercalated *A*WO_4_ Scheelite-Type Tungstates

**DOI:** 10.3390/ma17133071

**Published:** 2024-06-22

**Authors:** Georgijs Bakradze, Edmund Welter, Alexei Kuzmin

**Affiliations:** 1 Institute of Solid State Physics, University of Latvia, 8 Kengaraga Street, LV-1063 Riga, Latvia; 2 Deutsches Elektronen-Synchrotron—A Research Centre of the Helmholtz Association, Notkestrasse 85, D-22607 Hamburg, Germany; edmund.welter@desy.de

**Keywords:** tungstate, polaron, EXAFS, reverse Monte Carlo, DFT, LCAO

## Abstract

The studies of polaronic centers in a homologous series of scheelite-type compounds *A*WO_4_ (*A* = Ca, Sr, Ba) were performed using the W L_3_-edge and Sr K-edge X-ray absorption spectroscopy combined with the reverse Monte Carlo simulations, X-ray photoelectron spectroscopy (XPS), and first-principles calculations. Protonated scheelites H*_x_A*WO_4_ were produced using acid electrolytes in a one-step route at ambient conditions. The underlying mechanism behind this phenomenon can be ascribed to the intercalation of H^+^ into the crystal structure of tungstate, effectively resulting in the reduction of W^6+^ to W^5+^, i.e., the formation of polaronic centers, and giving rise to a characteristic dark blue-purple color. The emergence of the W^5+^ was confirmed by XPS experiments. The relaxation of the local atomic structure around the W^5+^ polaronic center was determined from the analysis of the extended X-ray absorption fine structures using the reverse Monte Carlo method. The results obtained suggest the displacement of the W^5+^ ions from the center of [W^5+^O_4_] tetrahedra in the structure of *A*WO_4_ scheelite-type tungstates. This finding was also supported by the results of the first-principles calculations.

## 1. Introduction

Polarons in transition metal oxides and related compounds represent a widespread phenomenon and strongly influence their physical properties and functionality [1,2,3,4]. Polaron forms when a strong electron-phonon interaction leads to the displacement of atoms from their equilibrium positions to screen the electron charge [5]. In the case of tungsten oxides, the appearance of the polaronic state is responsible for the optical absorption band with an energy of about 0.6–0.8 eV [6].

Many transition metal oxides exhibit electrochromism, i.e., a property to reversibly and stably change their optical properties under an applied electric field [7,8,9]. This property makes them good candidates for applications in various electronic and optoelectronic devices [10,11,12]. Electrochromism is caused by a pairwise charge insertion/extraction of small ions—typically H^+^ or Li^+^—and electrons to respect the overall charge electroneutrality. These electrons—when localized at the metal sites—change the metal oxidation state and polarize the lattice around, leading to the formation of polaronic states [13,14]. Tungsten trioxide (WO_3_) is probably the most well known and studied electrochromic material [7], which finds practical applications in smart windows and mirrors [9,11]. Note that radiation-induced color centers [WO_4_]^3−^—created by γ- or X-ray irradiation—have been also observed in several tungstates, including CaWO_4_ and MgWO_4_ [15,16,17].

Tungstates of divalent metal ions, with the general formula *A*^2+^WO_4_, constitute a large class of functional materials finding different applications. Depending on the size of the *A* cation, these tungstates can crystallize in either the wolframite structure (monoclinic crystal system, space group P2/c, no. 13, Z=2) or the scheelite structure (tetragonal crystal system, space group $I4_{1}/a$, no. 88, Z=2) [18]. The formal oxidation state of W in *A*^2+^WO_4_ tungstates is 6+, but it can be reduced either by changing the stoichiometry or by the insertion of protons. Such behavior is well known in pure tungsten oxides WO_3−x_, where it is responsible for the oxide photochromic and electrochromic properties and has been investigated over many decades [6,7,14,19]. In particular, X-ray absorption spectroscopy (XAS) has previously been employed to investigate changes in the local structure caused by polaron formation upon proton intercalation in amorphous tungsten trioxide thin films [20].

Although extensively studied in WO_3_ [21,22], the detailed structural effects of proton intercalation in scheelite-type tungstates *A*^2+^WO_4_ remain underexplored. In this study, we addressed this gap by performing the first direct structural study of the polaronic centers in a series of isomorphous scheelite-type tungstates, such as CaWO_4_, SrWO_4_, and BaWO_4_ by XAS. The local structure relaxation around tungsten ions induced by localized electrons was determined from the analysis of extended X-ray absorption fine structure (EXAFS) spectra using the reverse Monte Carlo (RMC) method and compared with the results of first-principles density functional theory (DFT) calculations and X-ray photoelectron spectroscopy.

## 2. Materials and Methods

### 2.1. Sample Synthesis and Characterization

*A*WO_4_ (*A* = Ca, Sr, Ba) tungstates were produced using a co-precipitation method with stoichiometric amounts of aqueous solutions of Na_2_WO_4_·2H_2_O (99+%, Alfa Aesar, Haverhill, MA, USA) and metal nitrates (Ca(NO_3_)_2_·4H_2_O (≥99.0%, Sigma-Aldrich, St. Louis, MO, USA), Sr(NO_3_)_2_·6H_2_O (≥99%, Sigma-Aldrich, St. Louis, MO, USA), Ba(NO_3_)_2_·6H_2_O (≥99%, Sigma-Aldrich, St. Louis, MO, USA)). The synthesis was performed at room temperature and pH = 8, followed by annealing at 550 °C in air. WO_3_·H_2_O powder was obtained by adding hydrochloric acid to aqueous solution of Na_2_WO_4_·2H_2_O as described in [23,24] and was used for comparison. The phase purity of all samples was confirmed by powder X-ray diffraction (XRD) at room temperature. The XRD measurements were performed using a benchtop Rigaku MiniFlex 600 diffractometer (Rigaku, Tokyo, Japan) with a Bragg–Brentano geometry. The X-ray tube with a copper anode (Cu K*α* radiation) operated at 40 kV and 15 mA.

A protonation of tungstates was performed using the method employed in the past for tungsten oxides [25,26]. For each sample, 500 mg of tungstate powder and 200 mg of metallic In wire were immersed in a 1.0 mL HCl aqueous solution with varying concentrations (0.5, 1.5, and 3.0 mol/L, respectively). The degree of protonation was controlled by adjusting the acid concentrations. Subsequently, the suspensions were ultrasonicated for 30 min. After removing the indium pieces, the mixtures were centrifuged, and the precipitates were collected and thoroughly washed in isopropanol. Finally, they were dried on a hot plate at 60 °C. Three powder samples of each tungstate were selected for XAS experiments: pristine (white, i.e., uncolored) samples, intermediate light-blue colored samples, and dark-blue/purple colored samples. As a quantitative assessment of the actual degree of protonation was not possible, we therefore refer to these samples qualitatively as pristine, light-blue colored, and dark blue-purple colored samples, respectively.

X-ray photoelectron spectroscopy (XPS) of the tungstate powder samples was carried out at room temperature using a Thermo Scientific ESCALAB Xi+ system (Waltham, MA, USA) with monochromatic Al K*α* radiation (hν = 1486.68 eV, 14.8 kV, 175 W). The binding energy scale was calibrated according to the method described in [27]. The binding energy values of the recorded spectra were charge-corrected by setting the alkyl C 1s peak binding energy position to 285.0 eV. This typically required shifting the recorded spectra by approximately 1.0 eV for pristine samples and 0.1 eV for colored samples. The integral area of the C 1s peak never exceeded 5% of that of the most intense photoelectron peak in the survey.

### 2.2. X-ray Absorption Spectroscopy and Data Analysis

XAS measurements were conducted at the DESY PETRA-III P65 undulator beamline (Hamburg, Germany) [28]. The storage ring operated at *E* = 6.08 GeV and *I* = 120 mA in a top-up 480 bunch mode. Harmonic rejection was accomplished using two uncoated plane mirrors (for W L_3_-edge) and Rh-coated mirrors (for Sr K-edge). The X-ray absorption spectra were collected using a fixed-exit Si(111) monochromator in transmission mode at the W L_3_-edge 10,207 eV) for *A*WO_4_ (*A* = Ca, Sr, Ba) tungstates and, in addition, at the Sr K-edge (16,105 eV) for SrWO_4_. The X-ray intensity was monitored using three ionization chambers. The first chamber was filled with nitrogen gas, whereas the second one with a mixture of nitrogen and argon gases. The N_2_/Ar gas ratio in the second chamber was optimized for each absorption edge. The third chamber was always filled with krypton gas and used to measure the reference tungsten foil for energy calibration.

The measurements were performed at 10 K using the Janis Research Company, LLC (Woburn, MA, USA) liquid helium flow cryostat to reduce thermal disorder. Powder samples were milled in an agate mortar, deposited onto Millipore filters and secured with Scotch tape. The sample weight was adjusted to achieve absorption edge jumps of approximately 1.

**Figure 1:**
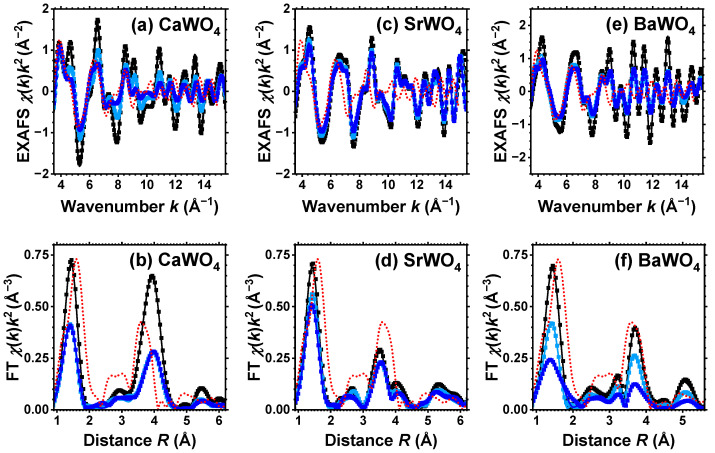
Experimental (symbols) and RMC/EA-simulated (solid lines) W L_3_-edge EXAFS spectra $\chi \left(k\right)k^{2}$ (**upper** panels) and their Fourier transforms (FTs) (**lower** panels) at 10 K for CaWO_4_ (**a**,**b**), SrWO_4_ (**c**,**d**), and BaWO_4_ (**e**,**f**). Black, light blue, and blue curves correspond to pristine (white), light-blue colored, and dark blue-purple colored samples, respectively. Only moduli of FTs are shown. Red dotted lines show experimental data for the W L_3_-edge in WO_3_·H_2_O. See text for details.

**Figure 2:**
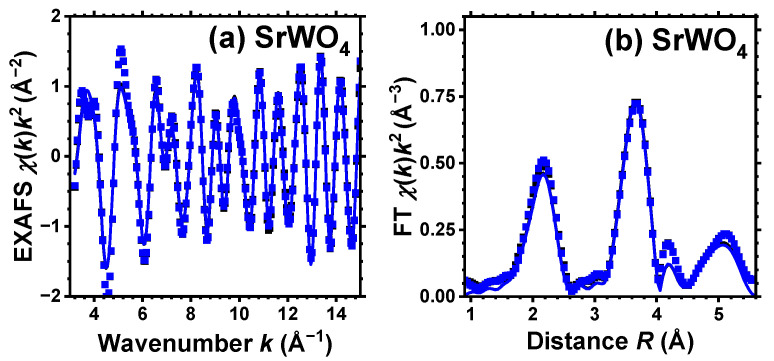
Experimental (symbols) and RMC/EA-simulated (solid lines) Sr K-edge EXAFS spectra $\chi \left(k\right)k^{2}$ (**a**) and their Fourier transforms (FTs) (**b**) at 10 K for SrWO_4_. Black and blue curves correspond to pristine (white) and dark blue-purple colored samples, respectively, and they almost coincide. Only the moduli of FTs are shown. See text for details.

The conventional procedure [29] as implemented in the XAESA code [30] was used to extract the EXAFS spectra $\chi \left(k\right)k^{2}$ from X-ray absorption spectra. The photoelectron wavenumber *k* is defined as $k=\sqrt{\left(2m_{e}/\hbar^{2}\right)\left(E-E_{0}\right)}$, where *E* is the X-ray photon energy, $E_{0}$ is the core electron binding energy, $m_{e}$ is the electron mass, and *ℏ* is the reduced Planck’s constant. A comparison of multiple measurements conducted on each sample revealed that the statistical noise in the EXAFS spectra was significantly lower than the systematic uncertainty stemming from data processing. The extracted EXAFS spectra and their Fourier transforms (FTs) at the W L_3_-edge and Sr K-edge are shown, respectively, in Figure 1 and Figure 2 by symbols. Note that all reported FTs are not adjusted for the backscattering phase shift of atoms. Consequently, all peak positions are shifted towards smaller distances compared to their crystallographic values.

The presence of the expected distortion of the local atomic structure in tungstates upon polaron formation complicates the analysis of EXAFS spectra by conventional methods. Therefore, in this study, the analysis is performed using the RMC method with an evolutionary algorithm (EA), which is implemented in the EvAX code [31]. The method was successfully employed in the past to examine several compounds featuring the scheelite structure [32] and to investigate local lattice distortions surrounding impurities [33].

In our RMC/EA approach, initial structural models for both pristine and protonated scheelites were constructed as 4a×4b×2c supercells with the periodic boundary conditions based on the known crystallographic structures. The lattice parameters *a*, *b*, and *c* were obtained from the XRD data of CaWO_4_ [34], SrWO_4_ [34], and BaWO_4_ [34]. Throughout the RMC/EA simulation process, the shape and size of the supercells remain unchanged [35,36].

During each step of the RMC/EA simulation, all atoms within the supercell underwent random displacement to accommodate both static and dynamic disorder. The maximum allowable displacement from the ideal crystallographic position was set at 0.4 Å to be larger than the expected structural relaxations and thermal disorder effects. The convergence of the RMC simulation was accelerated using the evolutionary algorithm [31]. The process was repeated until the difference between the Morlet wavelet transforms (WT) [37] of the experimental and configuration-averaged (CA) EXAFS $\chi \left(k\right)k^{2}$ spectra was minimized simultaneously in *k* and *R* space.

CA-EXAFS spectra were computed at every step of the RMC/EA simulation, employing the ab initio real-space multiple-scattering (MS) approach and the complex exchange correlation Hedin–Lundqvist potential [38] with the FEFF8.5L code [39,40,41]. The cluster potential calculations were conducted within the muffin-tin (MT) self-consistent-field approximation, utilizing the default MT radii values implemented in the FEFF8.5L code [39].

For pristine and protonated SrWO_4_, a single structural model was used to fit simultaneously both the EXAFS spectra acquired at the W L_3_-edge and Sr K-edge (see Figure 1 and Figure 2). However, for CaWO_4_ and BaWO_4_, the structural model was solely fitted to the W L_3_-edge EXAFS spectrum. In *k*-space, the fit ranges were 3.0–15.0 Å^−1^ for the Sr K-edge and 3.5–16.0 Å^−1^ for the W L_3_-edge. In *R*-space, the fit range was 1.0–5.5 Å for both edges. The results of the RMC/EA simulations for the W L_3_-edge and for the Sr K-edge are presented in Figure 1 and Figure 2, respectively. Note that only two spectra—for pristine (white) and dark blue-purple colored samples—are shown for clarity at the Sr K-edge, and they are almost identical.

**Figure 3:**
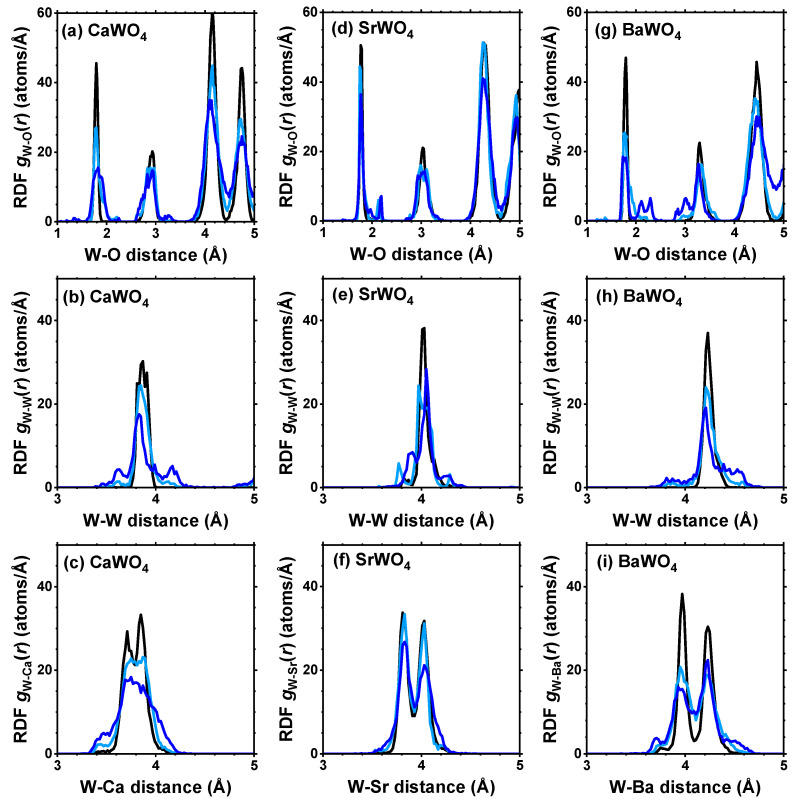
Radial distribution functions (RDFs) $g_{W–O}\left(r\right)$, $g_{W–W}\left(r\right)$, and $g_{W–A}\left(r\right)$ (*A* = Ca, Sr, Ba) in polycrystalline pristine (black) and protonated—intermediate blue colored (light blue) and dark blue-purple colored (blue)—(**a**–**c**) CaWO_4_, (**d**–**f**) SrWO_4_, and (**g**–**i**) BaWO_4_ at 10 K obtained using the RMC/EA simulations. See text for details.

**Figure 4:**
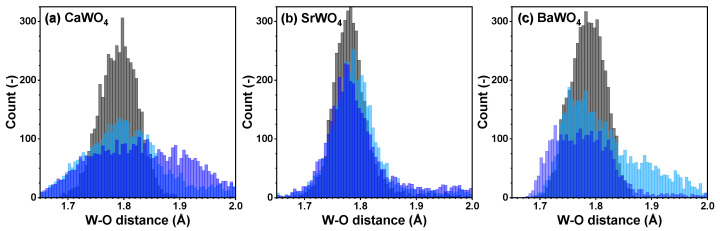
Histograms of the W–O distances for the nearest group of oxygen atoms—cf. radial distribution functions (RDFs) $g_{W–O}\left(r\right)$—in polycrystalline pristine (black) and two protonated—intermediate blue colored (light blue) and dark blue-purple colored (blue)—(**a**) CaWO_4_, (**b**) SrWO_4_, and (**c**) BaWO_4_ samples at 10 K obtained using the RMC/EA simulations. See text for details.

**Figure 5:**
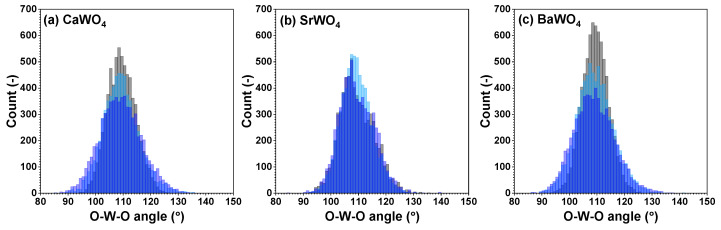
Angular distribution functions for intra-tetrahedral angles O–W–O within [WO_4_] tetrahedra in polycrystalline pristine (black) and two protonated—intermediate blue colored (light blue) and dark blue-purple colored (blue)—(**a**) CaWO_4_, (**b**) SrWO_4_, and (**c**) BaWO_4_ samples at 10 K obtained using the RMC/EA simulations. See text for details.

**Figure 6:**
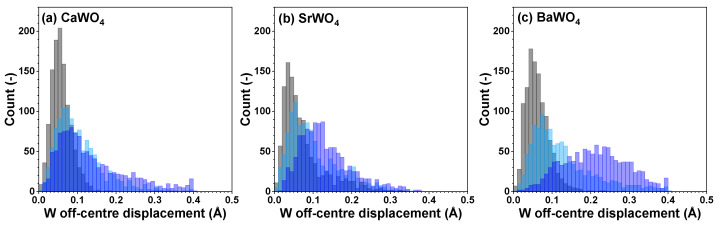
Distributions of off-center displacements of W within [WO_4_] tetrahedra in polycrystalline pristine (black) and two protonated—intermediate blue colored (light blue) and dark blue-purple colored (blue)—(**a**) CaWO_4_, (**b**) SrWO_4_, and (**c**) BaWO_4_ samples at 10 K obtained using the RMC/EA simulations. See text for details.

From each RMC/EA simulation, a structural model—comprising a set of atomic coordinates—was obtained. This model was then employed to compute the partial radial distribution function (RDF) for tungsten–oxygen atom pairs, the angular distribution function (ADF) for intra-tetrahedral angles O–W–O, and the distribution of off-center displacements of tungsten in [WO_4_] tetrahedra. The RMC/EA simulations were repeated nine times starting from identical initial atomic configurations (as derived from the XRD data) but using different series of pseudo-random numbers to improve the statistics. The averaged RDFs $g_{W–O}\left(r\right)$, $g_{W–W}\left(r\right)$, and $g_{W–A}\left(r\right)$ (*A* = Ca, Sr, Ba) are shown in Figure 3. RDFs $g_{W–O}\left(r\right)$ for the nearest four oxygen atoms located in the first coordination shell of tungsten are depicted in Figure 4 in the form of histograms. The averaged ADFs for the O–W–O angles and the distributions of off-center displacements of tungsten in [WO_4_] tetrahedra are shown in Figure 5 and Figure 6, respectively, for pristine and two protonated tungstates.

### 2.3. First-Principles DFT Calculations

In order to explore the impact of hydrogen intercalation on the local structure of scheelite-type tungstates, first-principles calculations were conducted using the DFT linear combination of atomic orbitals (LCAO) method, implemented in the CRYSTAL17 code [42]. The basis sets selected for hydrogen, oxygen, and calcium atoms consisted of all-electron triple-zeta valence basis sets supplemented by one set of polarization functions [43]. In the case of heavy atoms (Sr, Ba, and W), the core electrons were excluded from consideration using the effective core pseudopotentials [44,45].

The Coulomb and exchange series were evaluated with the accuracy controlled by a set of tolerances, which were selected to be (10^−8^, 10^−8^, 10^−8^, 10^−8^, and 10^−16^) as in our previous work [46], where these values allowed us to reproduce the atomic and electron structure of tungstates, as well as the pressure-induced phase transitions. The Brillouin zone was integrated using the Monkhorst–Pack scheme [47] over an 8 × 8 × 8 *k*-point mesh. Self-consistent field calculations were conducted utilizing the M06 [48] functional with a tolerance of 10^−4^ for the total energy change.

First, calculations were performed for the tetragonal space group *I*4_1_/*a* (no. 88) with two formula units (12 atoms) in the primitive cell, starting from the crystallographic structure [49,50,51]. A reasonable agreement between the calculated and experimental values of the lattice parameters (*a* and *c*) and the atomic fractional coordinates (*x*, *y*, *z*) of the oxygen atom was found (Table 1). The charges of ions were also estimated from the Mulliken population analysis. Note that their values deviate from the formal ionic charges due to the partial covalent character of metal–oxygen bonding.

Next, calculations were carried out for a primitive cell enlarged by 2 × 2 × 2 (a supercell) and containing 97 atoms: 16 *A* (*A* = Ca, Sr, or Ba), 16 W, 64 O, 1 H. In this case, all point group symmetry operators were removed, and one hydrogen atom was added to the supercell and placed next to one of the oxygen atoms. The optimization of atom coordinates and cell parameters involved minimizing the total energy. While the model used is rather simple, it enabled us to mimic the primary changes occurring in the structure of scheelites upon hydrogen intercalation.

**Figure 7:**
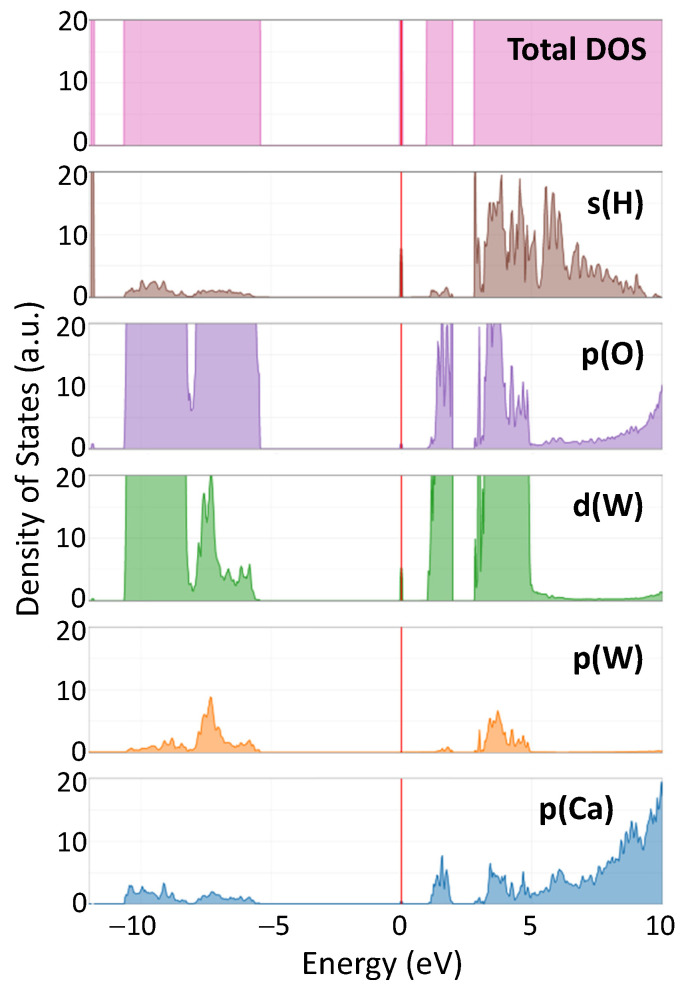
Calculated total and projected density of states (DOS) in protonated CaWO_4_. The vertical red line indicates the position of the Fermi level.

Figure 7 shows the total density of states (DOS) in hydrogen-doped CaWO_4_, along with the DOS projected onto individual atomic orbitals (s(H), p(O), d(W), p(W), p(Ca)). The Fermi level, which represents the energy reference point, is set at the position of the hydrogen impurity band. The states in the valence band are located at negative energies, whereas those in the conduction band are at positive energies.

**Figure 8:**
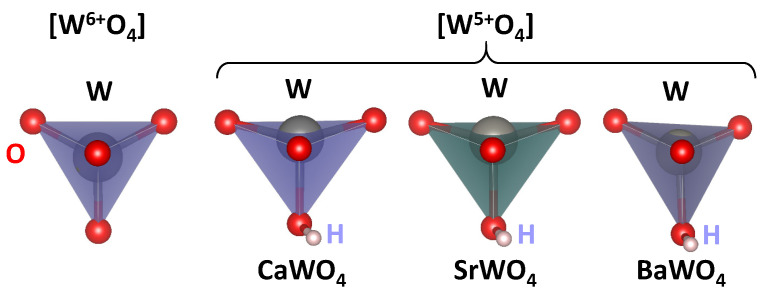
Regular [W^6+^O_4_] and distorted [W^5+^O_4_] tetrahedra in CaWO_4_, SrWO_4_, and BaWO_4_ according to the DFT LCAO calculations. Hydrogen (H) bound to the one of oxygen atoms is also shown.

The calculated lattice parameters *a* and *c*, atomic fractional coordinates of the oxygen atom (*x*, *y*, *z*), interatomic distances $R_{W–O}$, and atomic charges estimated from the Mulliken population analysis [52] for pristine scheelites are reported in Table 1. The average values of the interatomic distances $\langle R_{W–O}\rangle$ for undistorted [W^6+^O_4_] and distorted [W^5+^O_4_] tetrahedra, bond lengths in distorted [W^5+^O_4_] tetrahedra, and Mulliken charges *q* obtained from the results of the supercell calculations are given in Table 2. The undistorted (regular) [W^6+^O_4_] and distorted [W^5+^O_4_] tetrahedra in three tungstates are shown in Figure 8.

## 3. Results and Discussion

Good-quality experimental EXAFS spectra for pristine and protonated *A*WO_4_ (*A* = Ca, Sr, Ba) samples were recorded at 10 K, allowing us to perform RMC/EA simulations to track the variations in the local environment around tungsten ions upon protonation. Because it was possible to obtain the Sr K-edge EXAFS spectrum across a broad energy span, SrWO_4_ was the only compound for which the EXAFS spectra of both Sr and W cations were collected and simultaneously used in the RMC/EA simulations. The results depicted in Figure 1 and Figure 2 demonstrate a good agreement between the experimental data (dots) and the EXAFS spectra calculated using the RMC/EA method (solid lines), as well as their Fourier transforms, both before and after protonation.

The first broad peak in the FTs of the W L_3_-edge EXAFS spectrum at about 1.5 Å in Figure 1 is attributed to the first coordination shell of tungsten, while the strong peak at about 3.8 Å predominantly arises from the metal atoms within the second coordination shell [32]. The effect of protonation is clearly apparent in the W L_3_-edge EXAFS spectra, resulting in the damping of the EXAFS oscillations at large *k*-values, indicating the occurrence of structural distortions. The degree of protonation affects the amplitude of all peaks in FTs, which diminishes as more protons enter the tungstate structure. The effect is most pronounced in CaWO_4_ and least pronounced in BaWO_4_.

As it is apparent from Figure 2, the effect of protonation, as expected, is hardly noticeable at the Sr K-edge for pristine and dark-blue colored SrWO_4_ samples. Indeed, their Sr K-edge EXAFS spectra and FTs show almost no significant changes at least up to 4 Å. This result indicates that the formation of polaronic color centers occurs at tungsten ions.

For the sake of comparison, Figure 1 also shows the experimental W L_3_-edge EXAFS spectrum and its FT for WO_3_·H_2_O. It is known [53,54,55] that scheelites dissolve in aqueous solutions of inorganic acids with the formation of the insoluble product WO_3_·H_2_O. However, as it is apparent from Figure 1, the EXAFS spectra from pristine and protonated scheelites do not show any similarity to the EXAFS spectrum of WO_3_·H_2_O. Thus, we conclude that under our experimental conditions, the formation of WO_3_·H_2_O does not occur in the amounts capable of altering the EXAFS results. However, we cannot exclude that WO_3_·H_2_O shells form on the surface of *A*WO_4_ grains.

The atomic configurations derived from the RMC/EA simulations are employed for computing partial RDFs $g_{W–O}\left(r\right)$, $g_{W–W}\left(r\right)$, and $g_{W–A}\left(r\right)$ (*A* = Ca, Sr, Ba) (Figure 2). As it is evident from Figure 3 (cf. with Figure 4), in all three tungstates, the first coordination shell of W, consisting of four oxygen atoms, becomes broader with increasing degree of protonation, indicating the occurrence of local distortions. The broadening effect is also well visible in the outer shells.

Because interatomic distances are not sensitive to angular distortions, we use atomic coordinates from final RMC simulation boxes to analyze the geometry of [WO_4_] coordination tetrahedra also in terms of the angular distribution functions for the intra-tetrahedral angle O–W–O (Figure 5). As can be seen from the widths of O–W–O angle distributions, in pristine state, [WO_4_] tetrahedra are more regular in BaWO_4_ than in the other two scheelites. Upon protonation, however, the largest distortion of [WO_4_] tetrahedra occurs in CaWO_4_ and BaWO_4_, whereas in SrWO_4_, the degree of distortion is smaller. The origin of such distortion is attributed to the off-center displacements of W atoms upon hydrogen intercalation (Figure 6). It should be noted, however, that considerable decoloration of protonated SrWO_4_ occurred much faster—often during the XAS sample preparation stage—than in protonated CaWO_4_ or BaWO_4_.

Thus, the results of the RMC/EA analysis suggest that in protonated scheelite-type tungstates, the local geometry around tungsten atoms deviates from regular tetrahedral coordination and depends on the degree of protonation.

The underlying mechanism behind the described observations can be explained by hydrogen intercalation into the crystal structure of scheelite-type tungstate in the form of H^+^ ions and electrons e^−^ as required by the electroneutrality condition. During this process, the proton intercalation sites are expected to be near the O^2−^ anions, while additional electrons become localized at central tungsten ions, resulting in a distortion of their local environment and the appearance of polaronic centers [13,14,19]. The localization of electron at the tungsten ions effectively modifies its oxidation state from W^6+^ to W^5+^, causing the material color to change from white to a characteristic dark blue color [56,57,58].

We use the DFT LCAO calculations to support our experimental findings and the proposed model of a polaronic center in scheelites. The results of the calculations suggest that the insertion of hydrogen into the scheelite structure results in the formation of an O–H bond with a typical bond length of about 0.99 Å and a reduction in the effective charge on the nearest tungsten ion (Table 1 and Table 2) from about *q*(W^6+^) = 2.75–2.79 to *q*(W^5+^) = 2.65–2.71. Additionally, the [W^5+^O_4_] tetrahedron becomes distorted, with one of the four W^5+^–O bonds being slightly longer than the other three (Table 2). The distortion can be characterized as a displacement of the tungsten ion from the center of the [WO_4_] tetrahedron (Figure 8). The theoretically predicted tungsten displacement is larger in CaWO_4_ and SrWO_4_ compared to BaWO_4_.

**Figure 9:**
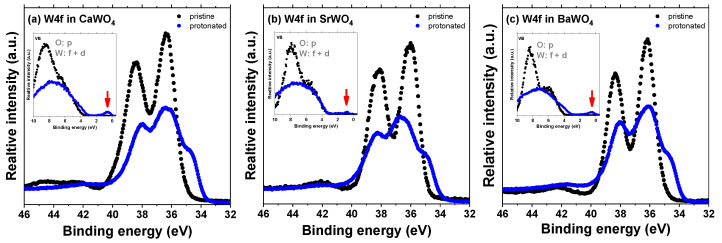
High-resolution X-ray photoelectron spectra of the W 4f region as recorded from pristine and protonated (**a**) CaWO_4_, (**b**) SrWO_4_, and (**c**) BaWO_4_ at the zero detection angle. The insets show the valence-band region. The red arrows point to a narrow band originating from the 1s(H) and 5d(W) states.

The impact of the hydrogen intercalation on the electronic structure of scheelites can be understood from the total density of states (DOS) of the supercell shown in Figure 7 for CaWO_4_. Upon intercalation, a narrow band originating from 1s(H) and 5d(W) states appears in the band gap at about 1 eV below the bottom of the conduction band (Figure 3). This band is similar to that found for the single-electron polaron in WO_3_ [59]. These states have been observed in the X-ray photoelectron spectra of the valence-band region in pristine and protonated scheelites (see red arrows in the insets in Figure 9). The XPS spectra of the W 4f region clearly indicate the presence of the W^5+^ ions in protonated samples: the W 4f signal is broadened towards lower energy, suggesting the appearance of tungsten ions with an oxidation state lower than 6+ [60,61]. Indeed, the doublet of 34.7 eV for W 4f_7/2_ and 36.8 eV for W 4f_5/2_ corresponding to the W^5+^ ions was observed in hydrogen rich WO_3_ films in [62]. A similar contribution from the W^5+^ ions was found in the XPS spectra of the W 4f core level in WO_3_ nanowire films after they were bombarded by an Ar_+_ ion beam [63].

The electrons located in this band are responsible for the optical absorption in the near infra-red spectral range as was found in tungsten oxides [13,14,64,65]. The autolocalization of free electrons at [WO_4_]^2−^ complex anions resulting in the creation of the [WO_4_]^3−^ centers was also demonstrated by electron spin-resonance (ESR) measurements in [66].

## 4. Conclusions

We conducted the first experimental and theoretical studies of polaronic centers in a series of isomorphous scheelite-type tungstates *A*WO_4_ (*A* = Ca, Sr, Ba) using X-ray absorption and photoelectron spectroscopies and DFT calculations. Protonated tungstates H*xA*WO_4_ were produced using acid electrolytes in a one-step route at ambient conditions.

The increasing proton concentration has a clear effect on the local structure of protonated scheelite-type tungstates, which was evidenced by a significant change in the shape of the W L_3_-edge EXAFS spectra and the amplitude of all peaks in their FTs. At the same time, it was found that the Sr K-edge EXAFS in SrWO_4_ remains almost unchanged during protonation.

The RMC/EA analysis [31] of EXAFS spectra was used to reveal the detailed variations in the local atomic structure of tungstates upon proton intercalation. We found that an increasing degree of protonation leads to a static distortion of the [WO_4_] tetrahedra due to the off-center displacements of W^5+^ atoms, whose existence was confirmed by XPS experiments. The effect of protonation on the local geometry of tetrahedral [WO_4_] units is greater in BaWO_4_ and CaWO_4_ compared to SrWO_4_.

A microscopic model of polaronic centers in scheelite-type tungstates is proposed and supported by the DFT LCAO calculations. The simultaneous insertion of a proton and electron into the scheelite structure results in electron localization on the nearest tungsten ion, leading to a reduction in its charge and off-center displacement, and the formation of the O–H bonds. The process is accompanied by the appearance of a narrow band due to 1s(H) and 5d(W) states located at about 1 eV below the bottom of the conduction band and being responsible for the dark blue-purple color. This band is similar to that found for the single-electron polaron in WO_3_ [59].

To conclude, we have experimentally revealed the effect of protonation on the atomic structure of scheelite-type tungstates through the analysis of EXAFS spectra using the reverse Monte Carlo method. A microscopic model of the structure of polaronic centers in tungstates has been proposed.

## Figures and Tables

**Table 1:** Experimental [49,50,51] and DFT LCAO calculated values of the lattice parameters (*a* and *c*), the fractional coordinates (*x*, *y*, *z*) of the oxygen atom, and the energy band gap $E_{g}$ in tetragonal *A*WO_4_ (*A* = Ca, Sr, Ba) scheelites with the space group *I*4_1_/*a* (no. 88, Z=2).

	CaWO_4_		SrWO_4_		BaWO_4_	
	**Experiment**	**LCAO**	**Experiment**	**LCAO**	**Experiment**	**LCAO**
*a* (Å)	5.2429	5.27	5.4168	5.47	5.6149	5.64
*c* (Å)	11.3737	11.34	11.951	11.88	12.7326	12.48
*x*(O)	0.1507	0.157	0.2497	0.231	0.2295	0.226
*y*(O)	0.0086	0.012	0.3425	0.105	0.1294	0.116
*z*(O)	0.2106	0.211	0.1671	0.042	0.05024	0.045
*R*(W–O) (Å)	1.78	1.79	1.78	1.79	1.78	1.79
*q*(*A*^2+^)		1.67		1.95		1.32
*q*(W^6+^)		2.79		2.77		2.75
*q*(O^2−^)		−1.11		−1.18		−1.02

**Table 2:** The average values of the interatomic distances 〈R〉(W–O), the bond lengths *R*(W^5+^–O*N*) (*N* = 1, 2, 3, or 4 is the oxygen atom number) in [W^5+^O_4_] tetrahedra, and Mulliken charges *q* obtained from the results of the DFT LCAO calculations.

	CaWO_4_	SrWO_4_	BaWO_4_
〈R〉(W^6+^–O) (Å)	1.80	1.79	1.79
〈R〉(W^5+^–O) (Å)	1.89	1.88	1.85
*R*(W^5+^–O1) (Å)	1.84	1.82	1.79
*R*(W^5+^–O2) (Å)	1.85	1.84	1.79
*R*(W^5+^–O3) (Å)	1.85	1.85	1.84
*R*(W^5+^–O4) (Å)	2.01	2.01	1.98
*q*(W^5+^)	2.71	2.68	2.65
*q*(H^+^)	0.38	0.35	0.36

## Data Availability

Data associated with this article are available upon reasonable request to the authors because of ongoing research.

## Abbreviations

The following abbreviations are used in this manuscript:

ADF	angular distribution function
CA	configuration-averaged
DFT	density functional theory
DOS	density of states
EA	evolutionary algorithm
EXAFS	extended X-ray absorption fine structure
FT	Fourier transform
LCAO	linear combination of atomic orbitals
MS	multiple-scattering
MT	muffin-tin
RDF	radial distribution function
RMC	reverse Monte Carlo
WT	wavelet transform
XAS	X-ray absorption spectroscopy
XPS	X-ray photoelectron spectroscopy
XRD	X-ray diffraction
